# Socioeconomic and Ethnic Disparities in Malnutrition Among Children Under Six Years of Age in Vietnam's Poor Districts

**DOI:** 10.1111/mcn.70264

**Published:** 2026-10-21

**Authors:** Tu S. Nguyen, Tuan T. Nguyen, Bac H. Nguyen, Linh N. P. Hoang, Truong H. Nguyen, Anh N. P. Hoang, Phuong N. Huynh, Thao T. Nguyen, Duong T. Tran

**Affiliations:** ^1^ Department of General Planning National Institute of Nutrition Hanoi Vietnam; ^2^ College of Health Sciences VinUniversity Hanoi Vietnam; ^3^ Innovations in Health Sciences VinUniversity Hanoi Vietnam; ^4^ Department of School and Occupational Nutrition National Institute of Nutrition Hanoi Vietnam; ^5^ Directorial Office, National Institute of Nutrition Hanoi Vietnam; ^6^ Department of General Practice School of Public Health and Preventive Medicine, Monash University Melbourne Australia; ^7^ Scientific Management Division, National Institute of Nutrition Hanoi Vietnam; ^8^ School of Chemistry and Life Sciences Hanoi University of Science and Technology Hanoi Vietnam

**Keywords:** child malnutrition, ethnic disparities, poverty, socioeconomic inequalities, stunting, Vietnam

## Abstract

Despite progress in improving nutritional status in Vietnam, persistent disparities among ethnic minority populations and children in poor districts remain, underscoring the need for context‐specific evidence to inform equitable nutrition policies. This study assessed the prevalence and determinants of malnutrition among children under 6 years of age in poor districts of Vietnam. A cross‐sectional study was conducted among 7747 children aged 0–71 months and their mothers from ten poor districts selected through multistage random sampling. Anthropometric measurements and nutritional status were classified following WHO protocols. Survey‐adjusted logistic regression accounting for clustering by district and commune examined factors associated with stunting, underweight, wasting, and overweight. Nearly half of households were classified as poor (48.8%), and access to improved water and sanitation was limited. Ethnic minorities comprised 92.3% of participants, namely Hmong, Dao, Thai, and Ra Glai. The prevalence of stunting, underweight, wasting, and overweight was 40.1%, 21.4%, 5.7%, and 3.0%, respectively, with stunting exceeding high public‐health‐significance thresholds in all 10 districts. Substantial ethnic disparities were observed, with Hmong (aOR = 2.74) and Ra Glai (aOR = 2.11) children showing markedly higher odds of stunting than Kinh children, and Ra Glai children uniquely at higher risk of wasting (aOR = 3.27) and coexistence of stunting‐wasting (aOR = 8.47). Fewer than four antenatal care visits, low birthweight (< 2500 g), male sex, and older age were independently associated with higher odds of stunting and/or underweight, while birthweight ≥ 3000 g was protective. Child undernutrition remains highly prevalent in Vietnam's poor districts and is marked by persistent ethnic, geographic, and biological disparities, underscoring the need for targeted, equity‐focused nutrition interventions in this population.

## Summary

- Stunting remains very high in poor districts of Vietnam, especially between ages 1–2 years.
- Large disparities persist across ethnic groups and regions, with minority children disproportionately affected.
- Household poverty, lack of improved water and sanitation, limited antenatal care, and maternal agricultural work are major risk factors for malnutrition.
- Growth faltering begins early in life, underscoring the need to focus interventions on pregnancy and the first 1000 days.
- Overweight is uncommon, indicating that undernutrition continues to be the dominant challenge in these disadvantaged areas.

## Graphical abstract

Stunting affected 40.1% of children in Vietnam's poor districts and exceeded high public health significance thresholds across all districts. The burden was greatest among ethnic minority children, supporting targeted interventions during the first 1000 days of life.

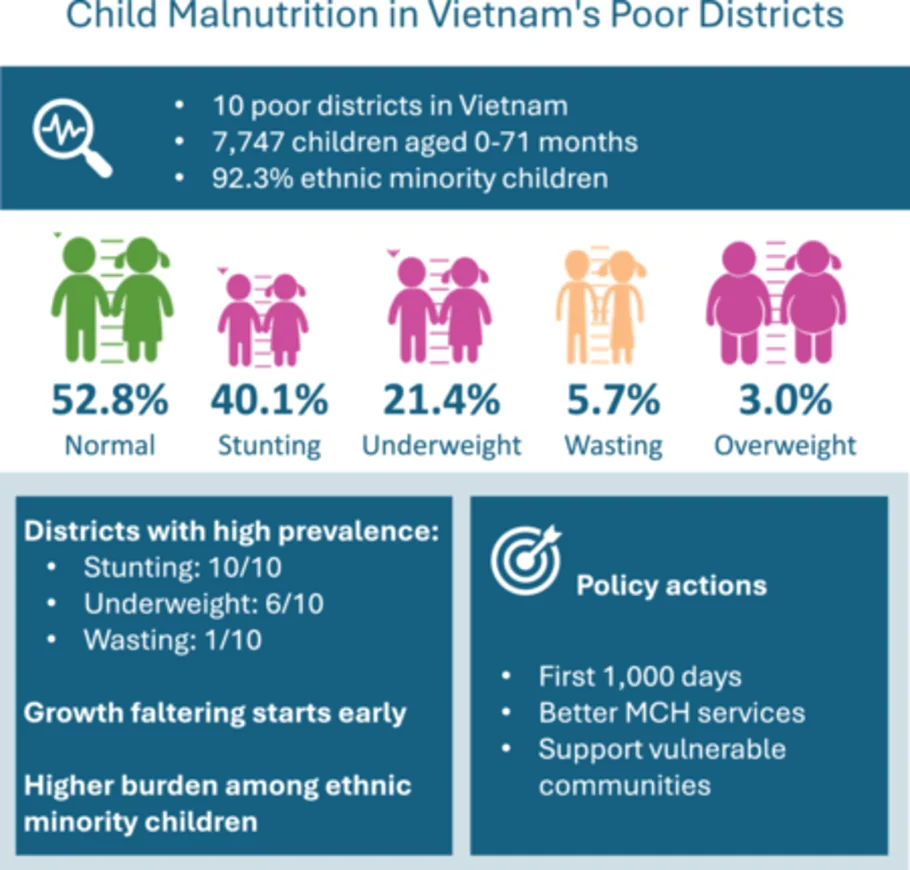

## 1 Introduction

Child malnutrition remains a critical global public health challenge, reflected in a triple burden of undernutrition (stunting, wasting, underweight), micronutrient deficiencies, and rising overnutrition (overweight and obesity). These forms of malnutrition have profound consequences for physical growth, cognitive development, immune function, and long‐term economic productivity (UNICEF 2019; WHO, UNICEF, and WB. 2023). Stunting alone affects millions of children under 5 worldwide, impairing brain development and perpetuating intergenerational cycles of poverty, while micronutrient deficiencies—often termed “hidden hunger”—can compromise health even in the absence of overt underweight (WHO, UNICEF, and WB. 2023).

Vietnam has achieved substantial declines in child undernutrition over recent decades, driven by effective national policies, economic growth, and investments in health and nutrition. National stunting prevalence among children under five decreased from approximately 56.5% in 1990 to 29.3% in 2010, 24.6% in 2015, and approximately 19.6% in 2020 (NIN 2023). These improvements align with Vietnam's progress toward Sustainable Development Goal (SDG) 2.2 and position the country as a regional leader in stunting reduction.

Despite these national gains, substantial inequalities remain. Undernutrition disproportionately affects rural, mountainous, and ethnic minority communities, where stunting rates often exceed 25%–30%. For instance, stunting prevalence reached 37.4% in the mountainous and midland regions of northern Vietnam (NIN 2023). Ethnic minority children face significantly elevated risks, with odds ratios up to six times greater than those of the Kinh majority (Tan et al. 2025). These disparities reflect persistent inequities in access to diverse diets, healthcare, sanitation, education, and nutrition‐specific and nutrition‐sensitive interventions. Children living in poor districts—particularly in the northern mountainous region, Central Highlands, and ethnic minority communities—experience heightened vulnerabilities due to geographic isolation, socioeconomic disadvantages, cultural dietary practices, and inadequate infrastructure (NIN 2023).

National data from the General Nutrition Survey (GNS) 2020 document a high prevalence of stunting and micronutrient deficiencies in mountainous and ethnic minority areas (NIN 2023). However, existing datasets often lack sufficient granularity to accurately reflect conditions in specific poor districts (Tan et al. 2025). While national surveys provide valuable aggregated and subgroup‐level analyses (e.g., by ethnicity, region, wealth quintile), critical gaps persist in district‐level indicators, qualitative determinants of disparities, updated dietary assessments in remote underserved areas, and evaluations of intervention impacts in high‐risk settings (Mondon et al. 2024). These limitations hinder the design of tailored, equity‐focused nutrition strategies amid ongoing socioeconomic transitions and evolving program implementation (Mondon et al. 2024; Tan et al. 2025).

Given Vietnam's nutrition transition, persistent vulnerabilities in poor districts, and the need to equitably achieve national and global nutrition targets, there is an urgent need for localized evidence on patterns and determinants of nutritional disparities. This cross‐sectional study aims to examine differences in the nutritional status of children aged 0–71 months residing in selected poor districts of Vietnam.

## 2 Methods

### 2.1 Study Setting

This cross‐sectional study was conducted in ten districts of Vietnam classified as poor or facing high levels of socioeconomic disadvantage, as designated under Decision No. 353/QD‐TTg (dated March 15, 2022) of the Prime Minister (Prime Minister of Vietnam 2022d). This decision approved a list of 74 poor districts across 26 provinces for the National Target Program on Sustainable Poverty Reduction in the 2021–2025 period. The selected ten districts reflect typical geographic, climatic, and ethnic diversity patterns: the northern provinces (Cao Bang, Bac Kan, Lai Chau, Son La, Dien Bien, Ha Giang) are predominantly mountainous with ethnic minority populations and cooler climates; Dak Nong belongs to the elevated Central Highlands with red basalt soils; and Quang Nam and Ninh Thuan are part of the narrower coastal central region with drier conditions in the south.

Vietnam demonstrates a strong commitment to reducing child malnutrition, particularly stunting, in mountainous, rural, poor, and ethnic minority areas through the National Nutrition Strategy 2021–2030 (Decision 02/QĐ‐TTg) and the National Target Programs (NTPs) for 2021‐2025, primarily the NTP for Socio‐Economic Development in Ethnic Minority and Mountainous Areas (Decision 1719/QĐ‐TTg) and the NTP for Sustainable Poverty Reduction (Decision 90/QĐ‐TTg) (Prime Minister of Vietnam 2022a, 2022b, 2022c, 2022d). These NTPs address stunting and other forms of malnutrition in poor households and disadvantaged areas, with a focus on mothers, children under 5, school‐age children, and adolescents, supported by multi‐sectoral efforts and dedicated funding. From 2026 onward, these priorities continue under the integrated NTP for New Rural Development, Sustainable Poverty Reduction, and Socio‐Economic Development in Ethnic Minority and Mountainous Areas (2026‐2035; Phase I: 2026‐2030), approved via Resolution 257/2025/QH15 and detailed in Decision 417/QĐ‐BNNMT (National Assembly of Vietnam 2025). This consolidated program avoids overlaps, enhances efficiency, prioritizes disadvantaged regions, and sustains nutrition improvement alongside goals such as raising rural incomes, promoting equitable development, and reducing stunting.

### 2.2 Study Design, Sampling, and Sample Size

This cross‐sectional study was conducted in districts classified as poor under Decision No. 353/QĐ‐TTg issued by the Prime Minister, which approved the list of 74 poor districts in Vietnam for the 2021–2025 period. Ten districts were randomly selected from this list. Within each selected district, all communes were listed, and three communes were randomly selected. Within each selected commune, a complete list of children aged 0–71 months was compiled, including both preschool attendees and children not attending school. In this study, districts served as survey strata, and communes served as primary sampling units (PSU).

Children were stratified into three age groups (0–23.9 months, 24–47.9 months, and 48–71 months). Systematic random sampling was then used to select approximately 100 children from each age group within each commune. Specifically, a random starting child was selected, and subsequent children were chosen using a fixed sampling interval calculated as the ratio of the total number of eligible children to the required sample size within each age stratum. In communes with fewer than 300 eligible children, all eligible children were included.

The sample size was calculated to provide estimates of nutritional status at the commune level. Based on an anticipated prevalence of 37.4% for stunting (NIN 2023), a 95% confidence level, and a precision of ± 6%, the minimum required sample size was estimated to be 250 participants using the single‐proportion formula. This was increased to 278 participants to account for an anticipated 10% non‐response rate. The prevalence estimate of 37.4% was selected because it represented the prevalence of stunting in the mountainous and midland regions of northern Vietnam and was the value closest to 50% among the reported regional prevalences of stunting, wasting, and underweight in Vietnam (NIN 2023). For operational feasibility and to ensure adequate representation across age groups, the target sample size was rounded to approximately 300 children per commune, corresponding to about 100 children in each age group.

Mothers or primary caregivers of the selected children were invited to participate. A total of 7747 children aged 0–71 months were enrolled in the study, equivalent to an average of 258 child‐caregiver dyads per commune.

### 2.3 Data Collection

The research was conducted between March 2023 and June 2025. Data collection was conducted by trained investigators using standardized protocols. The study population comprised children aged 0–71 months and their mothers who were permanent residents of the selected communes and provided written informed consent. Individuals with severe physical deformities, chronic illnesses such as cardiovascular disease or diabetes, acute infections including pneumonia or diarrhea, or mental disorders were excluded.

Trained interviewers interviewed mothers of the children using a structured questionnaire. Anthropometric measurements included weight and height. Weight was measured using TANITA electronic scales with an accuracy of ± 0.1 kg, and height or length was measured using UNICEF wooden boards with an accuracy of ± 0.1 cm. Children under 24 months were measured lying down, while older children and mothers were measured standing. Measurements were conducted in accordance with WHO standardized protocols.

Quality assurance measures included centralized training for investigators, daily calibration of equipment, and on‐site data checks for completeness and consistency. Discrepancies were corrected immediately, and double data entry and validation were performed before analysis.

### 2.4 Study Variables

The primary outcomes of interest were indicators of child nutritional status, assessed using the WHO 2006 Child Growth Standards and expressed as Z‐scores. These included weight‐for‐age (WAZ), height‐for‐age (HAZ), weight‐for‐height (WHZ), and BMI‐for‐age (BAZ, for older children). Children were classified as stunted when HAZ was < −2 standard deviations (SD), underweight when WAZ was < −2 SD, and wasted when WHZ was < −2 SD. Overweight and obesity were defined as WHZ or BAZ > +2 SD. In addition, children's nutritional status was classified into four mutually exclusive categories: coexistence of stunting and wasting, stunting only, wasting only, and neither stunting nor wasting.

Covariates included child‐, maternal‐, and household‐level characteristics. Child‐level variables comprised age (in months and categorized into six age groups: < 1 year, 1, 2, 3, 4, and 5 years), sex, number of antenatal care visits, place of birth, and birthweight where available.

Maternal characteristics included age, educational attainment, occupation (agriculture vs. other occupations, such as wage employment or small business), and ethnicity. Maternal ethnicity was classified into 11 ethnic groups (Kinh, Tay, Nung, Dao, Hmong, Thai, Ra Glai, Mnong, Co Tu, Giay, and Khang), while all remaining ethnicities were grouped as “Other.” The “Other” category comprised approximately 19 officially recognized ethnic groups in Vietnam that were not represented among the 11 major ethnic groups.

Household‐level variables included household economic status (poor, near‐poor, or non‐poor according to the Government of Vietnam classification), household size, source of drinking water, and household sanitation facilities (toilet ownership and type).

Variables were selected a priori based on the UNICEF conceptual framework for child malnutrition and evidence from previous studies conducted in Vietnam and other low‐ and middle‐income countries (UNICEF 2019).

Missing values were minimal for most variables (< 2%). The exception was birthweight, for which 19.1% of observations were unavailable, largely because 20.1% of the children were born at home, where birthweight measurements were often not recorded.

### 2.5 Data Management and Analysis

Data were entered using EpiData version 3.1 (The EpiData Association, Odense, Denmark). Anthropometric indices were generated using WHO AnthroPlus software (World Health Organization, Geneva, Switzerland), which applies the WHO Child Growth Standards and WHO 2007 Growth Reference. All statistical analyses were conducted using Stata version 19.5 (StataCorp LLC, College Station, TX, USA) and incorporated the complex survey design, with district specified as the stratum and commune specified as the PSU. Sampling weights were not incorporated because sampling probabilities and survey weights were not calculated as part of the original study design.

First, descriptive statistics were calculated and presented as means and standard deviations (SDs) for continuous variables and as frequencies and percentages for categorical variables. The analyses included: (1) general characteristics of households, mothers, and children; (2) distributions of anthropometric Z‐scores; and (3) stratified analyses of nutritional status by age, sex, district, and ethnicity. Prevalence estimates for stunting, underweight, wasting, and overweight were presented with 95% confidence intervals (CIs).

The public health significance of stunting, underweight, wasting, and overweight prevalence was classified according to the WHO/UNICEF prevalence thresholds proposed by de Onis et al. (2019). Stunting and underweight prevalences of ≥ 20% to < 30% were considered high, and those ≥ 30% very high; wasting prevalence of 5% to < 10% was considered medium, 10% to < 15% high, and ≥ 15% very high; and overweight prevalence of 5% to < 10% was considered high and ≥ 10% very high.

Second, survey‐adjusted logistic regression models were performed to examine factors associated with stunting, underweight, wasting, and overweight, adjusting for child‐, maternal‐, and household‐level characteristics.

Finally, as supplementary analyses, multinomial logistic regression models were used to estimate adjusted odds ratios (aORs) and corresponding 95% confidence intervals (CIs) for nutritional status categories. Children who were neither stunted nor wasted served as the reference group, and models were adjusted for covariates selected a priori. In addition, adjusted linear regression models were conducted to examine factors associated with anthropometric Z‐scores.

### 2.6 Ethical Considerations

The study was approved by an appropriate Ethics Committee of the Vietnam National Institute of Nutrition (Established under Decision No. 525/QĐ‐VDD dated 12 May 2023; Approval Decision No. 688/QLKH‐VDD dated 13 July 2023). Written informed consent was obtained from all participants. Confidentiality was ensured by anonymizing data, and participants were informed of their right to withdraw at any time. Mothers and children received small compensation for participation.

## 3 Results

### 3.1 Characteristics of the Participants

Nearly half of the 7747 children aged 0–71months lived in households classified as poor (48.8%), and two‐thirds lived in households with at least five members. Access to improved water and sanitation was limited, with only 14.1% using tap water and 34.8% having no toilet facility. Most mothers worked in agriculture (81.2%), and ethnic minority groups accounted for the large majority of participants (92.3%), particularly Hmong (26.7%), Dao (16.1%), Thai (13.0%), and Ra Glai (9.1%) (Table 1).

**Table 1:** General characteristics of the mothers and the households (*n* = 7747).

	*n*	%
Household characteristics		
Household Economy		
Poor	3778	48.8
Near‐poor	1085	14.0
Better‐off	2884	37.2
Number of People in Household		
2–4	2563	33.1
5–6	3381	43.6
7–8	1308	16.9
9–13 (max value)	495	6.4
Water sources for household use		
Spring/stream/pond/lake	5502	71.0
Covered dug well	510	6.6
Uncovered dug well	71	0.9
Rainwater	239	3.1
Borewell	549	7.1
Tap water	1089	14.1
Public faucets/tanks	23	0.3
Others	46	0.6
Ownership of Toilet		
No toilet	2697	34.8
Single‐compartment toilet	1583	20.4
Two‐compartment toilet	835	10.8
Septic or semi‐septic toilet	2632	34.0
Maternal characteristics		
Mother age (year ± SD)	7747	27.9 ± 6.3
15–19	511	6.6
20–24	2437	31.5
25–29	2229	28.8
30–34	1440	18.6
35–51 (max value)	1130	14.6
Maternal education level		
Primary level or below	2772	35.8
Junior high school level	2509	32.4
Highschool level	1801	23.3
Some college	665	8.6
Mother's Occupation		
Farming/agriculture	6288	81.2
Small business/services	145	1.9
Salaried employee	558	7.2
Freelance work	572	7.4
Homemaker/unemployed/student	178	2.3
Mother's Ethnicity		
Kinh	594	7.7
Tay	522	6.7
Nung	178	2.3
Dao	1250	16.1
Hmong	2071	26.7
Thai	1004	13.0
Ra Glai	705	9.1
Mnong	374	4.8
Co Tu	575	7.4
Giay	159	2.1
Khang	76	1.0
Others^a^	239	3.1

^a^ The “Other” category comprised approximately 19 officially recognized ethnic groups in Vietnam that were not included amongthe 11 ethnic groups listed above.

Among 7747 children, the mean age was 3.2 ± 1.6 years, and the distributions of age and sex were balanced. Most children were born in health facilities (78.8%), although 21.2% were born at home or elsewhere. Nearly one‐fifth had no recorded birthweight, and almost two‐thirds were born to mothers who attended fewer than four antenatal care visits (Table 2).

**Table 2:** Characteristics of children (*n* = 7747).

	*N*	%
Age (years ± SD)	7747	3.2 ± 1.6
0	968	12.5
1	1130	14.6
2	1268	16.4
3	1501	19.4
4	1590	20.5
5	1290	16.7
Sex		
Male	3937	50.8
Female	3810	49.2
Number of Antenatal Check‐ups		
Three or fewer	4905	63.3
Four or more	2842	36.7
Place of Birth		
Commune health station	855	11.0
Hospital	5251	67.8
At home or other	1641	21.2
Birth Mode		
Vaginal birth	6585	85.0
Cesarean birth	1162	15.0
Measured Birthweight		
Not recorded	1482	19.1
< 2500 g	563	7.3
2500–2999 g	2072	26.7
≥ 3000 g	3630	46.9

### 3.2 Disparities in Nutritional Status Among Children Under 6 Years

Among participating children, all three anthropometric Z‐scores (WAZ, HAZ, and WHZ/BAZ) were below the WHO reference median in both boys and girls across all age groups (Figure 1). Mean WAZ and HAZ declined during the first 2 years of life, with HAZ showing the most pronounced reduction. Partial recovery in HAZ was observed after age two, although values remained below WHO reference standards throughout childhood. WHZ/BAZ remained stable across age groups in both sexes (Figure 1).

**Figure 1:**
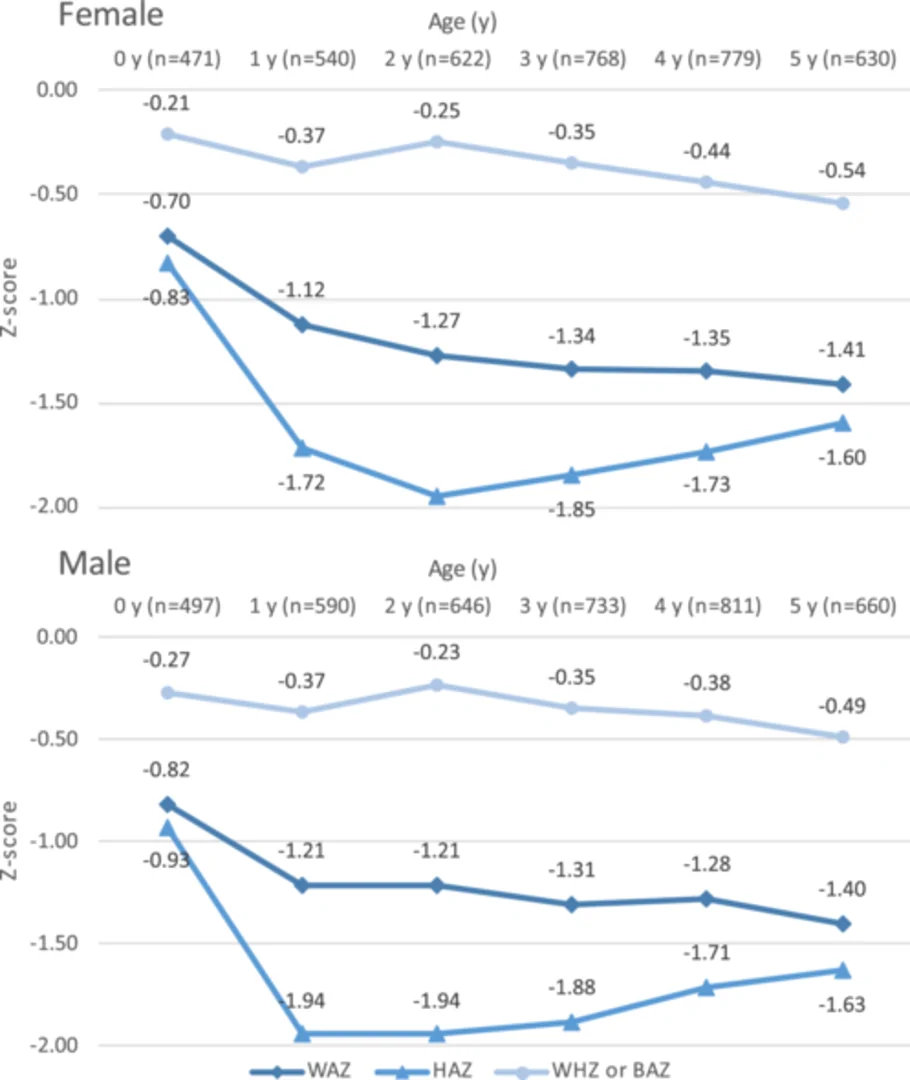
Z‐score nutritional status of children under 6 years of age by malnutrition type and age group (*n* = 7747). Abbreviations: weight‐for‐age (WAZ), height‐for‐age (HAZ), weight‐for‐height (WHZ for children aged 0–4 years), and BMI‐for‐age (BAZ for children aged 5 years).

The prevalence of stunting, underweight, wasting, and overweight was 40.1%, 21.4%, 5.7%, and 3.0%, respectively. Stunting prevalence increased from 20.4% among infants younger than 12 months to a peak of 47.3% at age 1 year and 48.3% at age 2 years before declining to 36.5% at age 5 years (Figure 2). Underweight prevalence increased from 13.3% among infants to 25.2% at age 5 years. Wasting was highest among infants (8.9%) and remained around 4% at ages 3–4 years, increasing to 6.6% at age 5 years. Overweight was uncommon, ranging from 1.3% to 5.2% across age groups. Similar age‐related patterns were observed among boys and girls (Figure 2).

**Figure 2:**
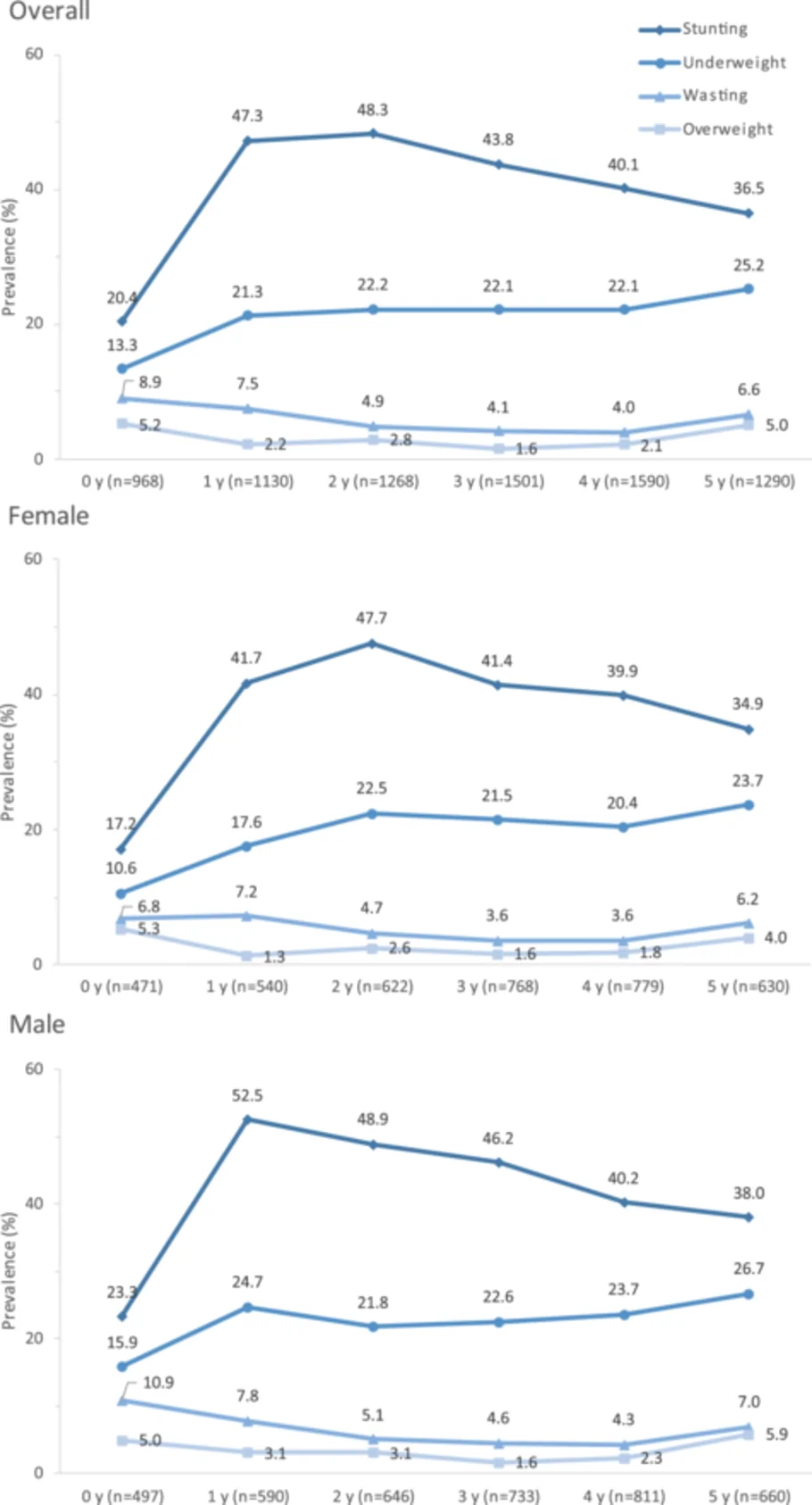
Nutritional status of children under 6 years of age by malnutrition type, sex, and age group (*n* = 7747).

Considerable geographic variation in malnutrition was observed across districts. Stunting exceeded the WHO threshold for high public health significance (≥ 20%) in all ten districts and surpassed very high prevalence (≥ 30%) in eight districts. Underweight prevalence was classified as high (≥ 20%) in six districts, while wasting prevalence was classified as high (≥ 10%) in one district (Figure 3).

**Figure 3:**
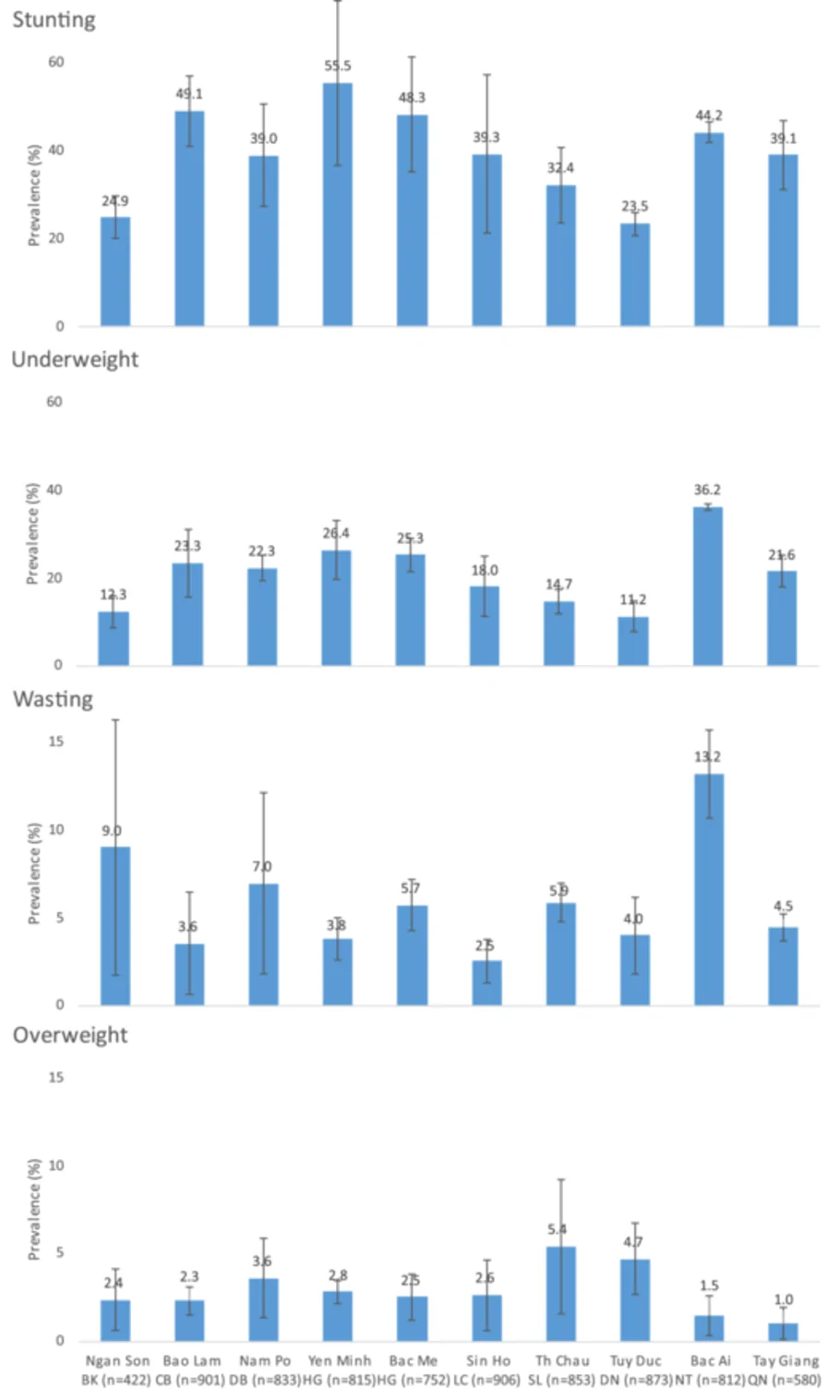
Prevalence of malnutrition among children under 6 years of age, by malnutrition type and district of residence (*n* = 7747). Values represent prevalence estimates and 95% confidence intervals (CIs).

Marked ethnic disparities in nutritional status were also evident. Stunting prevalence was classified as high (≥ 20%) in all ethnic groups and very high (≥ 30%) in nine groups. Underweight prevalence was classified as high (≥ 20%) in six ethnic groups and very high (≥ 30%) in one group. Wasting prevalence reached the high category (≥ 10%) only among Ra Glai children (14.0%) (Figure 4).

**Figure 4:**
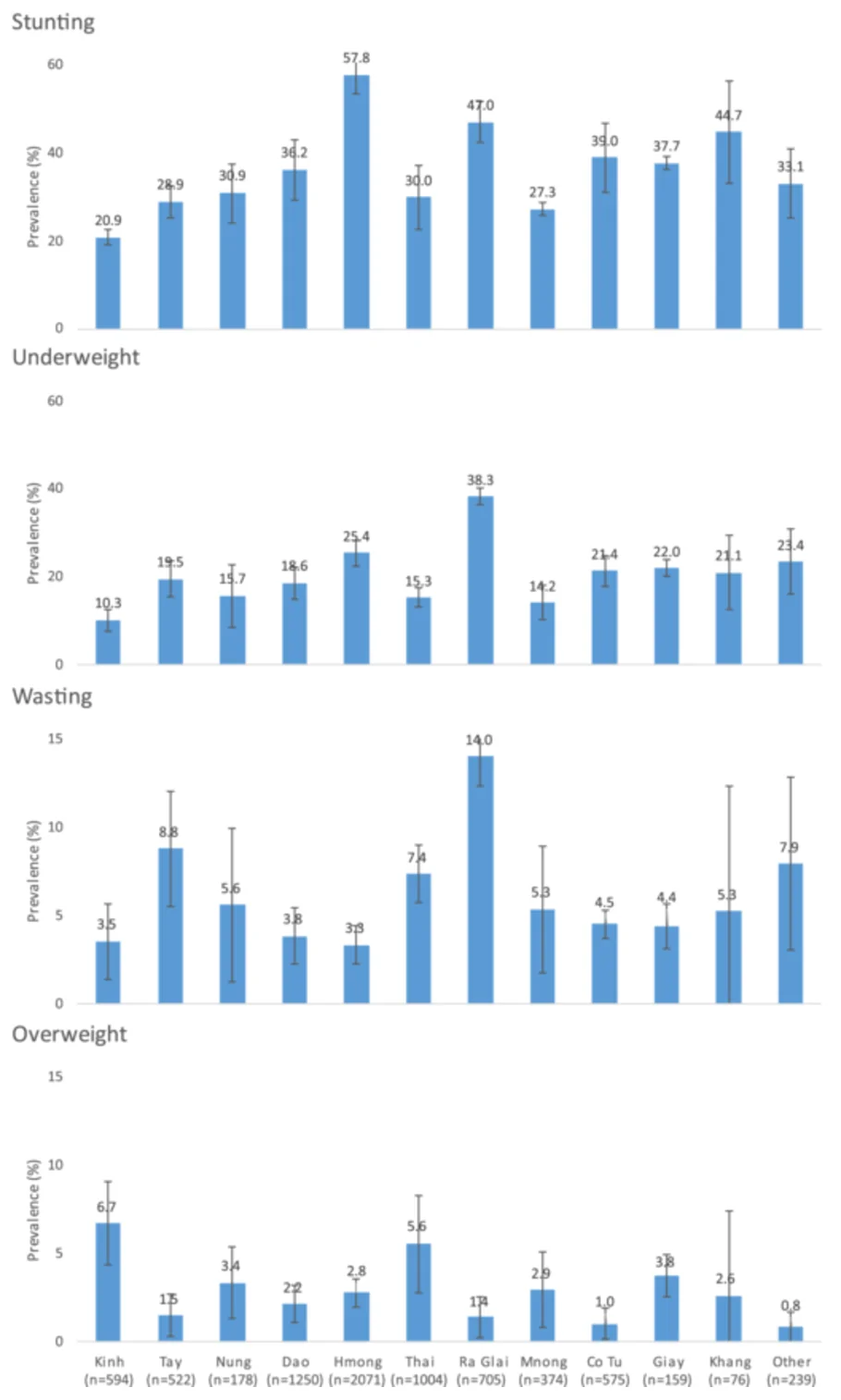
Prevalence of malnutrition among children under 6 years of age, by malnutrition type and maternal ethnicity (*n* = 7747). Values represent prevalence estimates and 95% confidence intervals (CIs).

### 3.3 Factors Associated With Stunting, Underweight, and Wasting

Table 3 presents factors associated with malnutrition among children under 6 years of age. Compared with children whose mothers were Kinh, children from six ethnic minority groups had significantly higher odds of stunting, particularly Hmong (aOR = 2.74; 95% CI: 1.96, 3.81) and Ra Glai children (aOR = 2.11; 95% CI: 1.32, 3.36). Elevated odds of stunting were also observed among Dao, Giay, Khang, and other minority ethnic groups. Underweight was significantly more common among Tay, Ra Glai, and other minority groups, while Ra Glai children were the only ethnic group with significantly higher odds of wasting (aOR = 3.27; 95% CI: 1.47, 7.24).

**Table 3:** Adjusted odds ratios (aORs) and 95% confidence intervals (CIs) for factors associated with stunting, underweight, wasting, and overweight among children under 6 years of age living in poor districts in Vietnam (*n* = 7747).

	Stunting	Underweight	Wasting	Overweight
A. Household characteristics				
Household economy				
Poor	1.14* (1.01, 1.29)	1.12 (1.00, 1.26)	1.08 (0.79, 1.48)	1 (0.76, 1.32)
Near‐poor	1.16 (0.99, 1.36)	1.13 (0.98, 1.31)	1.07 (0.73, 1.56)	0.83 (0.53, 1.30)
Better‐off	1 (1.00, 1.00)	1 (1.00, 1.00)	1 (1.00, 1.00)	1 (1.00, 1.00)
Households with ≥ 5 members	1.08 (0.96, 1.22)	1 (0.87, 1.15)	1.01 (0.84, 1.20)	0.9 (0.68, 1.18)
Not using tap water	1.13 (0.80, 1.59)	1.13 (0.95, 1.35)	1.15 (0.80, 1.65)	0.66* (0.47, 0.92)
Ownership of Toilet				
No toilet	1.17* (1.03, 1.34)	1.20* (1.04, 1.39)	0.99 (0.77, 1.27)	1.02 (0.73, 1.43)
Dry toilet (one or two tanks)	1.24** (1.08, 1.42)	1.02 (0.81, 1.27)	0.98 (0.71, 1.36)	1.14 (0.64, 2.03)
Septic or semi‐septic toilet	1 (1.00, 1.00)	1 (1.00, 1.00)	1 (1.00, 1.00)	1 (1.00, 1.00)
B. Maternal characteristics				
Mother age				
15–19	0.86 (0.68, 1.08)	1.17 (0.86, 1.58)	1.02 (0.66, 1.56)	0.96 (0.53, 1.76)
20–24	1 (1.00, 1.00)	1 (1.00, 1.00)	1 (1.00, 1.00)	1 (1.00, 1.00)
25–29	0.91 (0.80, 1.02)	0.9 (0.75, 1.07)	0.96 (0.69, 1.34)	0.94 (0.57, 1.54)
30–34	1.16** (1.04, 1.28)	1.01 (0.85, 1.19)	0.99 (0.74, 1.33)	1.15 (0.58, 2.30)
35–51	0.95 (0.80, 1.14)	1.01 (0.82, 1.23)	0.95 (0.61, 1.49)	1.18 (0.62, 2.26)
Mother ethnicities				
Kinh	1 (1.00, 1.00)	1 (1.00, 1.00)	1 (1.00, 1.00)	1 (1.00, 1.00)
Tay	1.16 (0.87, 1.54)	1.54* (1.05, 2.26)	1.97 (0.90, 4.34)	0.25* (0.09, 0.71)
Nung	1.22 (0.83, 1.80)	1.11 (0.54, 2.27)	1.28 (0.42, 3.89)	0.67 (0.31, 1.45)
Dao	1.50* (1.08, 2.10)	1.39 (0.90, 2.16)	0.89 (0.39, 2.00)	0.44* (0.21, 0.92)
Hmong	2.74*** (1.96, 3.81)	1.47 (0.93, 2.32)	0.67 (0.27, 1.64)	0.73 (0.41, 1.29)
Thai	1.24 (0.78, 1.98)	1.1 (0.71, 1.72)	1.76 (0.85, 3.66)	1.06 (0.54, 2.09)
Ra Glai	2.11** (1.32, 3.36)	3.33*** (2.30, 4.83)	3.27** (1.47, 7.24)	0.23** (0.08, 0.62)
Mnong	1.04 (0.82, 1.32)	1.11 (0.79, 1.54)	1.26 (0.42, 3.76)	0.62 (0.25, 1.55)
Co Tu	1.57 (0.89, 2.77)	1.48 (0.88, 2.50)	0.77 (0.36, 1.65)	0.17** (0.06, 0.49)
Giay	1.39* (1.02, 1.91)	1.45 (0.97, 2.18)	0.83 (0.38, 1.84)	0.78 (0.43, 1.43)
Khang	1.86* (1.10, 3.15)	1.18 (0.63, 2.23)	1.25 (0.25, 6.33)	0.73 (0.12, 4.59)
Others^a^	1.50* (1.07, 2.09)	2.03* (1.04, 3.99)	1.73 (0.56, 5.32)	0.13*** (0.04, 0.36)
Mother education level				
Primary level or below	1.24* (1.05, 1.46)	1.19* (1.02, 1.39)	1.14 (0.90, 1.46)	1.12 (0.76, 1.67)
Junior high school level	1 (1.00, 1.00)	1 (1.00, 1.00)	1 (1.00, 1.00)	1 (1.00, 1.00)
Highschool level	1.02 (0.80, 1.29)	1.16 (0.96, 1.41)	1.47 (0.95, 2.28)	1.39 (1.00, 1.93)
Some college	0.81 (0.60, 1.09)	0.93 (0.64, 1.35)	1.83* (1.16, 2.88)	2.18** (1.36, 3.49)
Occupation was a farmer	1.28** (1.12, 1.48)	1.27* (1.04, 1.56)	1.24 (0.88, 1.74)	1.06 (0.64, 1.75)
C. Child characteristics				
Antenatal checkup ≤ 3 times	1.29*** (1.17, 1.42)	1.35*** (1.18, 1.54)	1.23 (0.83, 1.82)	0.8 (0.62, 1.04)
Place of birth				
Not in a health facility	1.1 (0.92, 1.32)	1.2 (0.92, 1.57)	1.02 (0.53, 1.99)	0.82 (0.37, 1.85)
Commune health station	1.06 (0.88, 1.29)	1.1 (0.91, 1.33)	1.03 (0.71, 1.49)	0.97 (0.56, 1.68)
Hospital	1 (1.00, 1.00)	1 (1.00, 1.00)	1 (1.00, 1.00)	1 (1.00, 1.00)
Birthweight (g)				
Not recorded	0.87 (0.71, 1.08)	0.73 (0.53, 1.00)	0.9 (0.61, 1.33)	1.16 (0.60, 2.25)
< 2500 g	1.56** (1.18, 2.06)	1.69** (1.25, 2.29)	1.47* (1.09, 1.98)	1.19 (0.59, 2.40)
2500–2999 g	1 (1.00, 1.00)	1 (1.00, 1.00)	1 (1.00, 1.00)	1 (1.00, 1.00)
≥ 3000 g	0.58*** (0.50, 0.67)	0.52*** (0.45, 0.62)	0.62*** (0.50, 0.78)	1.04 (0.72, 1.51)
The child is a boy	1.37*** (1.27, 1.49)	1.31*** (1.18, 1.45)	1.25 (1.00, 1.58)	1.3 (0.97, 1.75)
Child age (years)				
0	1 (1.00, 1.00)	1 (1.00, 1.00)	1 (1.00, 1.00)	1 (1.00, 1.00)
1	4.01*** (3.02, 5.32)	1.83** (1.31, 2.55)	0.81 (0.52, 1.27)	0.39*** (0.24, 0.63)
2	4.49*** (3.17, 6.36)	2.13*** (1.64, 2.76)	0.54*** (0.43, 0.67)	0.44** (0.28, 0.71)
3	3.52*** (2.72, 4.57)	2.15*** (1.63, 2.83)	0.46** (0.29, 0.71)	0.24*** (0.14, 0.41)
4	3.06*** (2.30, 4.06)	2.18*** (1.61, 2.97)	0.44** (0.28, 0.69)	0.31*** (0.18, 0.52)
5	2.41*** (1.71, 3.39)	2.59*** (1.85, 3.62)	0.76 (0.50, 1.16)	0.78 (0.43, 1.41)

*Note:* aOR = adjusted odds ratio; CI = confidence interval. Survey‐adjusted subpopulation analysis.

* *p* < 0.05

** *p* < 0.01

*** *p* < 0.001.

^a^ The “Other” category comprised approximately 19 officially recognized ethnic groups in Vietnam that were not included among the 11 ethnic groups listed above.

Children whose mothers attended fewer than four antenatal care visits had higher odds of both stunting (aOR = 1.29; 95% CI: 1.17, 1.42) and underweight (aOR = 1.35; 95% CI: 1.18, 1.54). Low birthweight (< 2500 g) was associated with increased odds of stunting (aOR = 1.56; 95% CI: 1.18, 2.06), underweight (aOR = 1.69; 95% CI: 1.25, 2.29), and wasting (aOR = 1.47; 95% CI: 1.09, 1.98), whereas birthweight ≥ 3000 g was protective against all three forms of undernutrition (Table 3). Boys were more likely than girls to be stunted (aOR = 1.37; 95% CI: 1.27, 1.49) and underweight (aOR = 1.31; 95% CI: 1.18, 1.45). Age was also strongly associated with stunting and underweight, with children aged 1–5 years showing significantly higher odds of stunting and underweight than infants younger than 12 months (Table 3).

For additional analyses, the linear regression analyses of anthropometric Z‐scores demonstrated patterns consistent with those observed in the primary logistic regression models, particularly for ethnicity, sanitation, maternal occupation, antenatal care utilization, birthweight, and child age (Supporting Information S1: Table 1). In the multinomial logistic regression analysis, coexistence of stunting and wasting, representing the coexistence of chronic and acute undernutrition, was strongly associated with Ra Glai ethnicity (aOR = 8.47; 95% CI: 2.53, 28.36), low birthweight (aOR = 2.58; 95% CI: 1.59, 4.21), male sex (aOR = 1.64; 95% CI: 1.16, 2.32), and older child age compared with infants younger than 12 months (Supporting Information S1: Table 2).

## 4 Discussion

This study demonstrates that undernutrition remains a major public health challenge among children under 6 years of age in poor districts of Vietnam. Stunting was the most prevalent form of malnutrition, peaking at 1–2 years of age and remaining at levels of public health concern throughout early childhood. Substantial disparities were observed across districts and ethnic groups, with Hmong and Ra Glai children experiencing particularly high risks of undernutrition. Household poverty, inadequate sanitation, low maternal education, maternal agricultural occupation, limited antenatal care, and low birthweight were significantly associated with child nutritional status.

### 4.1 Burden and Pattern of Undernutrition

Recent nationally representative data indicate that although Vietnam has made steady progress in reducing child undernutrition, substantial subnational disparities persist. The GNS 2020 reported national prevalences of stunting (19.6%), underweight (11.5%), and wasting (4.2%) among children under 5 years of age (Tan et al. 2025; NIN 2023). Notably, these prevalences approached or exceeded WHO thresholds for public health significance, which classify stunting prevalence of 20– < 30% as high and ≥ 30% as very high, and wasting prevalence of 5– < 10% as medium and ≥ 10% as high (de Onis et al, 2019). This underscores the severity of undernutrition in the study districts.

We found early growth faltering in the first 1000 days, with deficits in linear growth emerging rapidly in infancy and persisting into early childhood. This pattern is consistent with extensive global and Vietnam‑specific evidence showing that growth faltering often begins in utero and accelerates during the first 2 years of life, reflecting the cumulative effects of maternal undernutrition, suboptimal infant feeding practices, recurrent infections, and poor living conditions (Leroy et al. 2014; Mertens et al. 2023). In Vietnam, longitudinal cohort studies have demonstrated that HAZ at 2 years is a strong predictor of later cognitive performance and mental health, highlighting the long‑term consequences of growth deficits established during this critical window (Nguyen et al. 2021). Our findings therefore reinforce the importance of prioritizing nutrition‑specific and nutrition‑sensitive interventions during the first 1000 days, including maternal nutrition before and during pregnancy, high‑quality antenatal care, optimal breastfeeding, appropriate complementary feeding, and infection prevention, particularly in deprived districts where early growth faltering remains highly prevalent.

### 4.2 Associated Factors During Pregnancy, at Birth, and Early Childhood

In this study, we found that children whose mothers attended fewer than four antenatal care visits were more likely to be stunted and underweight. Antenatal care provides opportunities for maternal nutritional assessment, micronutrient supplementation, management of pregnancy complications, and counseling on infant feeding and childcare practices. Similar associations between inadequate antenatal care and poorer child growth outcomes have been reported in multi‐country analyses and cohort studies from low‐ and middle‐income countries, highlighting the importance of strengthening maternal health services during pregnancy (Jiao et al. 2024; Hanieh et al. 2015).

Low birthweight emerged as one of the strongest predictors of undernutrition, being associated with increased odds of stunting, underweight, and wasting. In contrast, birthweight of at least 3000 g was protective against all three forms of undernutrition. These findings are consistent with previous evidence demonstrating that fetal growth restriction and adverse intrauterine conditions may contribute to growth deficits that may persist throughout early childhood and beyond (Hanieh et al. 2015; Leroy et al. 2014; Mertens et al. 2023). Furthermore, the results reinforce the importance of maternal nutrition and health interventions before and during pregnancy, as improvements in fetal growth and birth outcomes are likely to contribute to reducing childhood malnutrition and its long‐term consequences (Nguyen et al. 2021; Mertens et al. 2023).

Qualitative and quantitative studies from mountainous regions document culturally embedded feeding practices—such as delayed initiation of breastfeeding, discarding colostrum, prelacteal feeding, early complementary feeding, and low intake of animal‐source foods—that are correlated with higher rates of undernutrition (Huong and Nga 2013; Nguyen et al. 2016). Importantly, these practices are often shaped by poverty, food availability, and limited access to health information rather than tradition alone.

Dietary diversity remains markedly lower among ethnic minority households. Mixed‐methods studies among Hmong, Dao, and Thai communities show that children consume fewer vegetables, legumes, and protein‐rich foods than the national average, reflecting both limited market access and ecological constraints on food production (Nguyen et al. 2025; Mondon et al. 2024). Emerging evidence also indicates that ethnic minority children and adolescents are increasingly experiencing a double burden of malnutrition, with persistent undernutrition alongside rising overweight in settings undergoing rapid nutrition transition (Truong et al. 2022).

### 4.3 Maternal Characteristics Associated With Child Malnutrition

Disparities in maternal education and caregiving practices may contribute to intergenerational nutritional risk. Ethnic minority women are more likely to have low educational attainment, which has been consistently associated with poorer child growth outcomes in Vietnam (Beal et al. 2019).

We observed substantial ethnic disparities in nutritional status. Compared with Kinh children, Hmong and Ra Glai children experienced significantly higher odds of stunting, while Ra Glai children also had markedly higher odds of underweight and wasting.

This finding supports a growing body of peer‐reviewed literature indicating that ethnic disparities in child nutrition in Vietnam are not biologically determined but instead arise from intersecting structural, geographic, socioeconomic, and cultural factors. Recent national evidence shows that multidimensional child poverty affects 35.9% of ethnic minority children, compared with only 6.5% of Kinh/Hoa children, highlighting the substantial socioeconomic disadvantages faced by ethnic minority populations in Vietnam (GSO and UNICEF 2025).

Decomposition analyses confirm that household wealth explains the largest share of national stunting reduction; however, slow income growth and limited livelihood diversification among ethnic minorities have reduced their ability to benefit from economic development (Harris et al. 2021). Low‐quality water, sanitation, and hygiene (WASH) environments increase exposure to infections and environmental enteric dysfunction, undermining nutrient absorption and child growth (Bekele et al. 2020). Longitudinal evidence from Vietnam indicates that access to improved sanitation in infancy is associated with sustained reductions in stunting risk through middle childhood, with stronger effects than improved water access alone (Dearden et al. 2017). These patterns further disadvantage ethnic minority communities, where WASH infrastructure remains limited.

### 4.4 Socioeconomic and Environmental Factors

Most ethnic minorities reside in remote mountainous regions with limited infrastructure, market access, and public services. National survey analyses show that residence in mountainous areas independently increases the risk of stunting, even after controlling household socioeconomic status (Tan et al. 2025). Inadequate transport infrastructure and reduced availability of health and nutrition services further amplify these disadvantages (Beal et al. 2019). The geographic isolation of groups such as the Hmong, Ra Glai, and Khang may contribute to their very high stunting rates, as they predominantly reside in remote mountainous and forested highland regions where access to markets, diverse foods, and health services is limited (GSO and UNICEF 2025; Mbuya et al. 2019; UNICEF 2015). In addition, certain ethnic groups—particularly the Hmong—exhibit patterns of periodic mobility or shifting settlement linked to upland agricultural systems such as shifting cultivation, which may further disrupt consistent access to services (Nguyen et al. 2021).

Among the Hmong, extreme remoteness, language barriers, and limited proficiency in Vietnamese may further hinder access to essential health and nutrition services. Ra Glai communities experience similar terrain‑related constraints in the forested highlands, while Khang communities face geographic isolation in the northwestern region alongside subsistence‑based livelihoods that limit food security and service utilization. These systemic disparities persist despite national progress, underscoring the need for targeted, culturally responsive interventions tailored to the realities of these communities.

Finally, policy implementation gaps and limited cultural responsiveness act as structural contributors to ethnic inequalities. Although Vietnam has implemented targeted nutrition and poverty‐reduction programs (Prime Minister of Vietnam 2021, 2022a, 2022b, 2022c; Nguyen et al. 2023), interventions are often designed with uniform assumptions about language, access, and behavior, limiting their effectiveness in ethnic minority contexts (Harris et al. 2021). These findings underscore the need for culturally adapted, locally tailored nutrition strategies that align with the social, ecological, and economic realities of ethnic minority communities (GSO and UNICEF 2025).

### 4.5 Strengths and Limitations

This study has several strengths. It draws on a large and diverse sample of 7747 children under 6 years of age from ten nationally designated poor districts, providing strong statistical power and ensuring relevance to socioeconomically and geographically disadvantaged populations. The inclusion of districts from northern mountainous areas, the Central Highlands, and central coastal regions enabled meaningful assessment of regional and ethnic disparities in child nutritional status. Anthropometric measurements were collected by trained field staff using standardized WHO protocols, with rigorous quality control, ensuring high measurement validity and comparability with national and international datasets. In addition, the study adopted a comprehensive analytical framework by examining multiple forms of malnutrition—including stunting, wasting, underweight, and their concurrence—thereby capturing the complex and overlapping nature of child undernutrition.

This study has limitations as well. The cross‑sectional design limits causal interpretation, and relationships between household, maternal, and child characteristics and nutritional outcomes should be interpreted as associations. Key variables, including household economic status and antenatal care use, were self‑reported, which may be subject to recall or social desirability bias. Although survey‐adjusted analyses were used to account for the multistage sampling design, sampling weights were not available because sampling probabilities were not calculated as part of the original survey design. Therefore, while the findings provide important evidence from the selected poor districts, prevalence estimates may not be fully representative of the broader population of children living in poor districts across Vietnam. Furthermore, as the study focused specifically on poor districts, results may not be generalizable to urban or wealthier populations in Vietnam. Finally, unmeasured contextual factors, such as seasonal food availability, caregiving constraints, and local variations in program implementation, may have influenced the observed associations between the explanatory variables and child nutritional status.

## 5 Conclusions and Recommendations

Despite substantial national progress, child malnutrition remains a major public health challenge in poor districts of Vietnam, particularly during the first 3 years of life. Stunting, underweight, and wasting were associated with inadequate sanitation, low maternal education, ethnic minority status, limited antenatal care, and low birthweight.

To accelerate progress toward Vietnam's nutrition targets, interventions should prioritize the first 1000 days of life, strengthen maternal and child health services, improve household living conditions, and implement culturally appropriate nutrition programs for ethnic minority communities.

## Author Contributions

Conception or design of the work: Tu S. Nguyen, Tuan T. Nguyen, Duong T. Tran. Data collection: Tu S. Nguyen, Bac H. Nguyen, Linh N. P. Hoang. Data analysis and interpretation: Tu S. Nguyen, Tuan T. Nguyen. Graphical design: Tuan T. Nguyen. Drafting the article: Tu S. Nguyen, Tuan T. Nguyen. Providing critical feedback to improve the manuscript: all authors; revising the article: Tu S. Nguyen, Tuan T. Nguyen. All authors have reviewed the submitted version of the manuscript.

## Consent

Written informed consent was obtained from all participants.

## Conflicts of Interest

The authors declare no conflicts of interest.

## Supporting information


**Table S1:** Linear regression: adjusted coefficients (β) and 95% CI for child anthropometric Z‐scores.
**Table S2:** Factors associated with coexistence of stunting and wasting in children under six years of age (n = 7747).

## Data Availability

The data that support the findings of this study are available on request from the first or corresponding author. The data are not publicly available due to privacy or ethical restrictions. The data used and/or analyzed during the current study are available from the corresponding author based on a reasonable request. Requests for data may be directed to the first author and are subject to institutional data use agreements.

## References

[mcn70264-bib-0001] Beal, T. , D. T. Le , T. H. Trinh , et al. 2019. “Child Stunting Is Associated With Child, Maternal, and Environmental Factors in Vietnam.” Maternal & Child Nutrition 15, no. 4: e12826. 10.1111/mcn.12826. PMC6859968 30958643

[mcn70264-bib-0002] Bekele, T. , P. Rawstorne , and B. Rahman . 2020. “Effect of Water, Sanitation and Hygiene Interventions Alone and Combined With Nutrition on Child Growth in Low‐ and Middle‐Income Countries: A Systematic Review and Meta‐Analysis.” BMJ Open 10, no. 7: e034812. 10.1136/bmjopen-2019-034812. PMC7359184 32660947

[mcn70264-bib-0003] Dearden, K. A. , W. Schott , B. T. Crookston , D. L. Humphries , M. E. Penny , and J. R. Behrman , Young Lives Determinants and Consequences of Child Growth Project Team . 2017. “Children With Access to Improved Sanitation but Not Improved Water Are at Lower Risk of Stunting Compared to Children Without Access: A Cohort Study in Ethiopia, India, Peru, and Vietnam.” BMC Public Health 17: 110. 10.1186/s12889-017-4033-1. PMC5259877 28114914

[mcn70264-bib-0006] GSO and UNICEF . 2025. Disparities and Multidimensional Child Poverty among Ethnic Minority Populations in Viet Nam: Evidence From the 2024 Survey on the Socio‐economic Situation of 53 Ethnic Minorities. UNICEF Viet Nam. https://www.unicef.org/vietnam/documents/disparities-and-multidimensional-child-poverty-among-ethnic-minority-populations-viet-nam.

[mcn70264-bib-0007] Hanieh, S. , T. T. Ha , A. M. De Livera , et al. 2015. “Antenatal and Early Infant Predictors of Postnatal Growth in Rural Vietnam: A Prospective Cohort Study.” Archives of Disease in Childhood 100, no. 2: 165–173. 10.1136/archdischild-2014-306328. PMC4316936 25246090

[mcn70264-bib-0008] Harris, J. , P. Huynh , H. T. Nguyen , et al. 2021. “Nobody Left Behind? Equity and the Drivers of Stunting Reduction in Vietnamese Ethnic Minority Populations.” Food Security 13: 803–818. 10.1007/s12571-021-01183-7.

[mcn70264-bib-0009] Huong, L. T. , and V. T. T. Nga . 2013. “Nutritional Practices Among Ethnic Minorities and Child Malnutrition in Mountainous Areas of Central Vietnam.” Food and Nutrition Sciences 04: 82–89. 10.4236/fns.2013.41012.

[mcn70264-bib-0010] Jiao, B. , I. Iversen , R. Sato , et al. 2024. “Association Between Achieving Adequate Antenatal Care and Health‐Seeking Behaviors: A Study of Demographic and Health Surveys in 47 Low‐ and Middle‐Income Countries.” PLoS Medicine 21, no. 7: e1004421. 10.1371/journal.pmed.1004421. PMC11226092 38968303

[mcn70264-bib-0011] Leroy, J. L. , M. Ruel , J. P. Habicht , and E. A. Frongillo . 2014. “Linear Growth Deficit Continues to Accumulate Beyond the First 1000 Days in Low‐ and Middle‐Income Countries: Global Evidence From 51 National Surveys.” Journal of Nutrition 144, no. 9: 1460–1466. 10.3945/jn.114.191981. 24944283

[mcn70264-bib-0012] Mbuya, N. V. N. , S. J. Atwood , and P. N. Huynh . 2019. Persistent Malnutrition in Ethnic Minority Communities of Vietnam: Issues and Options for Policy and interventions. World Bank. 10.1596/978-1-4648-1432-7.

[mcn70264-bib-0013] Mertens, A. , J. Benjamin‐Chung , J. M. Colford , et al. 2023. “Causes and Consequences of Child Growth Faltering in Low‐Resource Settings.” Nature 621: 568–576. 10.1038/s41586-023-06501-x. PMC10511328 37704722

[mcn70264-bib-0014] Mondon, C. , P. Y. Tan , C. L. Chan , T. N. Tran , and Y. Y. Gong . 2024. “Prevalence, Determinants, Intervention Strategies and Current Gaps in Addressing Childhood Malnutrition in Vietnam: A Systematic Review.” BMC Public Health 24: 960. 10.1186/s12889-024-18419-8. PMC10996139 38575928

[mcn70264-bib-0015] National Assembly of Vietnam . 2025. Resolution No. 257/2025/QH15 Approving the Investment Policy for the National Target Program on New Rural Development, Sustainable Poverty Reduction, and Socio‐economic Development in Ethnic Minority and Mountainous Areas 2026–2035. https://thuvienphapluat.vn/phap-luat/toan-van-nghi-quyet-2572025qh15-ve-xay-dung-nong-thon-moi-giam-ngheo-va-phat-trien-vung-dan-toc-thi-247328.html.

[mcn70264-bib-0016] Nguyen, L. T. T. , M. van den Berg , T. Stomph , and D. Nabuuma . 2025. “Do Nutrition‐Sensitive Agriculture Interventions Work Among Ethnic Minorities in Northern Vietnam Amidst the COVID‐19 Crisis?” Food Security 17: 1153–1174. 10.1007/s12571-025-01580-2. PMC12549742 41142682

[mcn70264-bib-0017] Nguyen, P. H. , L. M. Tran , L. Q. Khuong , et al. 2021. “Child Linear Growth During and After the First 1000 Days Is Positively Associated With Intellectual Functioning and Mental Health in School‐Age Children in Vietnam.” Journal of Nutrition 151, no. 9: 2816–2824. 10.1093/jn/nxab182. PMC8417934 34113979

[mcn70264-bib-0018] Nguyen, T. A. , B. T. Nguyen , H. Van Ta , et al. 2021. “Livelihood Vulnerability to Climate Change in the Mountains of Northern Vietnam: Comparing the Hmong and the Dzao Ethnic Minority Populations.” Environment, Development and Sustainability 23, no. 9: 13469–13489. 10.1007/s10668-020-01221-y.

[mcn70264-bib-0019] Nguyen, T. T. , N. L. Huynh , P. N. Huynh , et al. 2023. “Bridging the Evidence‐To‐Action Gap: Enhancing Alignment of National Nutrition Strategies in Cambodia, Laos, and Vietnam With Global and Regional Recommendations.” Frontiers in Nutrition 10: 1277804. 10.3389/fnut.2023.1277804. PMC10800738 38260060

[mcn70264-bib-0020] Nguyen, T. T. , P. H. Nguyen , N. Hajeebhoy , H. V. Nguyen , and E. A. Frongillo . 2016. “Infant and Young Child Feeding Practices Differ by Ethnicity of Vietnamese Mothers.” BMC Pregnancy and Childbirth 16, no. 1: 214. 10.1186/s12884-016-0995-8. PMC4977888 27502920

[mcn70264-bib-0021] NIN . 2023. Findings of General Nutrition Survey 2019‐2020. Ministry of Health.

[mcn70264-bib-0022] Prime Minister of Vietnam . 2021. Decision No. 1719/QD‐TTg Approving the National Target Program on Socio‐economic Development in Ethnic Minority‐inhabited and Mountainous Areas in the 2021‐2030 Period, Phase I: From 2021 to 2025. https://vanban.chinhphu.vn/?pageid=27160&docid=204285.

[mcn70264-bib-0023] Prime Minister of Vietnam . 2022a. Decision 02/QD‐TTg Approving National Nutrition Strategy for the 2021–2030 Period With a Vision to 2045. https://vanban.chinhphu.vn/?docid=204983&pageid=27160.

[mcn70264-bib-0024] Prime Minister of Vietnam . 2022b. Decision No. 263/QD‐TTg Approving the National Target Program for New Rural Development 2021–2025. https://vanban.chinhphu.vn/?pageid=27160&docid=205378.

[mcn70264-bib-0025] Prime Minister of Vietnam . 2022c. Decision No. 90/QD‐TTg Approving the National Target Program for Sustainable Poverty Reduction 2021–2025. https://vanban.chinhphu.vn/?pageid=27160&docid=205180.

[mcn70264-bib-0026] Prime Minister of Vietnam . 2022d. Decision No. 353/QD‐TTg Approving the List of Poor Districts and Communes With Special Difficulties in Lowland Coastal Areas, Coastal Areas, and Islands for the 2021–2025 period. https://vanban.chinhphu.vn/?docid=205492&pageid=27160.

[mcn70264-bib-0027] Tan, P. Y. , S. V. Som , S. D. Nguyen , et al. 2025. “Demographic Variation and Socioeconomic Inequalities in All Forms of Malnutrition Among Children Aged 6 Months to 9 Years: Findings From the Vietnamese General Nutrition Survey 2020.” BMJ Public Health 3, no. 1: e001177. 10.1136/bmjph-2024-001177. PMC12320070 40761922

[mcn70264-bib-0028] Truong, D. T. T. , T. H. T. Tran , T. T. T. Nguyen , and V. H. T. Tran . 2022. “Double Burden of Malnutrition in Ethnic Minority School‐Aged Children Living in Mountainous Areas of Vietnam and Its Association With Nutritional Behavior.” Nutrition Research and Practice 16, no. 5: 658–672. 10.4162/nrp.2022.16.5.658. PMC9523200 36238380

[mcn70264-bib-0029] UNICEF . 2015. Nutrition in the Mountains of Viet Nam. UNICEF Viet Nam. https://unicefvietnam.blogspot.com/2015/11/nutrition-in-mountains-of-viet-nam.html.

[mcn70264-bib-0030] UNICEF . 2019. The State of the World's Children 2019: Children, Food and Nutrition: Growing Well in a Changing World. https://www.unicef.org/reports/state-of-worlds-children-2019.

[mcn70264-bib-0032] WHO, UNICEF, and WB . 2023. Levels and Trends in Child Malnutrition: UNICEF/WHO/World Bank Group Joint Child Malnutrition Estimates: Key Findings of the 2023 Edition. World Health Organization. https://iris.who.int/handle/10665/368038.

