# Supplementary material for: Socioeconomic and Ethnic Disparities in Malnutrition Among Children Under Six Years of Age in Vietnam's Poor Districts
Source: Matern Child Nutr. 2026 Oct 21;22(4):e70264. doi: 10.1111/mcn.70264 (PMC13686576; doi:10.1111/mcn.70264)
Supplement: Supplementary file 1 [file MCN-22-e70264-s001.docx]

**Supplementary Table 1.** Linear regression: adjusted coefficients (β) and 95% CI for child anthropometric Z-scores

|  | **Weight-for-age (WAZ)** | | **Height-for-age (HAZ)** | | **Weight-for-height (WHZ)** | |
| --- | --- | --- | --- | --- | --- | --- |
| **A. Household characteristics** |  |  |  |  |  |  |
| Household Economy |  |  |  |  |  |  |
| Poor | -0.03 | (-0.09, 0.02) | -0.07* | (-0.13, -0.01) | 0.02 | (-0.04,0.07) |
| Near-poor | -0.03 | (-0.09, 0.03) | -0.05 | (-0.11, 0.02) | -0.01 | (-0.10,0.08) |
| Better-off | 0 | (0.00, 0.00) | 0 | (0.00, 0.00) | 0 | (0.00,0.00) |
| Households with ≥ 5 members | -0.02 | (-0.07, 0.03) | -0.04 | (-0.10, 0.03) | 0.01 | (-0.05,0.06) |
| Not using tap water | -0.07** | (-0.12, -0.02) | -0.09 | (-0.22, 0.03) | -0.01 | (-0.13,0.11) |
| Ownership of Toilet |  |  |  |  |  |  |
| No toilet | -0.10** | (-0.17, -0.03) | -0.10* | (-0.18, -0.02) | -0.06* | (-0.12,-0.00) |
| Dry toilet (one or two tanks) | -0.07 | (-0.17, 0.03) | -0.18*** | (-0.26, -0.11) | 0.07 | (-0.04,0.17) |
| Septic or semi-septic toilet | 0 | (0.00, 0.00) | 0 | (0.00, 0.00) | 0 | (0.00,0.00) |
|  |  |  |  |  |  |  |
| **B. Maternal characteristics** |  |  |  |  |  |  |
| Mother age |  |  |  |  |  |  |
| 15-19 | -0.07 | (-0.16, 0.02) | -0.09 | (-0.20, 0.03) | -0.04 | (-0.15,0.07) |
| 20-24 | 0 | (0.00, 0.00) | 0 | (0.00, 0.00) | 0 | (0.00,0.00) |
| 25-29 | 0.03 | (-0.03, 0.09) | 0.02 | (-0.05, 0.09) | 0.02 | (-0.07,0.11) |
| 30-34 | -0.01 | (-0.08, 0.06) | -0.04 | (-0.11, 0.03) | 0.02 | (-0.08,0.12) |
| 35-51 | -0.01 | (-0.09, 0.08) | 0.01 | (-0.09, 0.10) | -0.02 | (-0.12,0.08) |
| Mother ethnicities |  |  |  |  |  |  |
| Kinh | 0 | (0.00, 0.00) | 0 | (0.00, 0.00) | 0 | (0.00,0.00) |
| Tay | -0.35*** | (-0.46, -0.23) | -0.09 | (-0.21, 0.03) | -0.44*** | (-0.61,-0.27) |
| Nung | -0.21* | (-0.40, -0.02) | -0.16 | (-0.36, 0.05) | -0.20* | (-0.38,-0.01) |
| Dao | -0.30*** | (-0.42, -0.18) | -0.30** | (-0.48, -0.12) | -0.18*** | (-0.26,-0.09) |
| Hmong | -0.32*** | (-0.45, -0.19) | -0.59*** | (-0.78, -0.41) | 0.06 | (-0.07,0.18) |
| Thai | -0.15 | (-0.33, 0.02) | -0.14 | (-0.33, 0.05) | -0.11 | (-0.25,0.04) |
| Ra Glai | -0.73*** | (-0.91, -0.55) | -0.45** | (-0.78, -0.12) | -0.66*** | (-0.78,-0.54) |
| Mnong | -0.21** | (-0.34, -0.09) | -0.09 | (-0.30, 0.12) | -0.25*** | (-0.39,-0.12) |
| Co Tu | -0.40** | (-0.62, -0.18) | -0.36** | (-0.58, -0.14) | -0.28** | (-0.45,-0.11) |
| Giay | -0.36*** | (-0.47, -0.25) | -0.32*** | (-0.45, -0.18) | -0.24** | (-0.38,-0.09) |
| Khang | -0.27*** | (-0.39, -0.14) | -0.38** | (-0.63, -0.12) | -0.06 | (-0.22,0.11) |
| Others† | -0.43*** | (-0.65, -0.20) | -0.27** | (-0.42, -0.11) | -0.39** | (-0.65,-0.12) |
| Mother education level |  |  |  |  |  |  |
| Primary level or below | -0.05 | (-0.11, 0.02) | -0.10* | (-0.19, -0.01) | 0.02 | (-0.06,0.10) |
| Junior high school level | 0 | (0.00, 0.00) | 0 | (0.00, 0.00) | 0 | (0.00,0.00) |
| Highschool level | 0.01 | (-0.06, 0.07) | 0.02 | (-0.08, 0.12) | 0 | (-0.08,0.07) |
| Some college | 0.1 | (-0.05, 0.25) | 0.15 | (-0.06, 0.36) | 0.01 | (-0.11,0.14) |
| Occupation was a farmer | -0.14*** | (-0.20, -0.08) | -0.12* | (-0.21, -0.03) | -0.10** | (-0.15,-0.05) |
| Antenatal checkup ≤3 times | -0.15*** | (-0.20, -0.11) | -0.18*** | (-0.24, -0.12) | -0.06 | (-0.12,0.01) |
|  |  |  |  |  |  |  |
| **C. Child characteristics** |  |  |  |  |  |  |
| Place of birth |  |  |  |  |  |  |
| Not in a health facility | -0.05 | (-0.12, 0.03) | -0.08 | (-0.17, 0.00) | 0.01 | (-0.10,0.13) |
| Commune health station | -0.10* | (-0.18, -0.02) | -0.07 | (-0.20, 0.05) | -0.07 | (-0.16,0.01) |
| Hospital | 0 | (0.00, 0.00) | 0 | (0.00, 0.00) | 0 | (0.00,0.00) |
| Birthweight |  |  |  |  |  |  |
| Not recorded | 0.11* | (0.01, 0.22) | 0.08 | (-0.02, 0.19) | 0.1 | (-0.01,0.20) |
| < 2500 g | -0.23*** | (-0.34, -0.11) | -0.23*** | (-0.35, -0.11) | -0.11 | (-0.25,0.03) |
| 2500–2999 g | 0 | (0.00, 0.00) | 0 | (0.00, 0.00) | 0 | (0.00,0.00) |
| ≥ 3000 g | 0.36*** | (0.30, 0.41) | 0.34*** | (0.25, 0.43) | 0.22*** | (0.16,0.28) |
| The child is a boy | -0.04 | (-0.08, 0.01) | -0.12*** | (-0.16, -0.08) | 0.01 | (-0.04,0.06) |
| Child age |  |  |  |  |  |  |
| 0 | 0 | (0.00, 0.00) | 0 | (0.00, 0.00) | 0 | (0.00,0.00) |
| 1 | -0.41*** | (-0.53, -0.30) | -0.97*** | (-1.13, -0.80) | -0.12 | (-0.29,0.05) |
| 2 | -0.54*** | (-0.64, -0.44) | -1.12*** | (-1.31, -0.93) | -0.04 | (-0.17,0.10) |
| 3 | -0.63*** | (-0.72, -0.53) | -1.03*** | (-1.17, -0.88) | -0.16* | (-0.31,-0.02) |
| 4 | -0.63*** | (-0.72, -0.54) | -0.90*** | (-1.04, -0.76) | -0.22** | (-0.38,-0.07) |
| 5 | -0.69*** | (-0.81, -0.58) | -0.75*** | (-0.93, -0.58) | -0.33*** | (-0.51,-0.16) |
| Constance | -0.16* | (-0.31, -0.01) | -0.13 | (-0.35, 0.08) | -0.02 | (-0.21,0.17) |
| Sample size | 7747 |  | 7747 |  | 7747 |  |

*Note: * p<0.05, ** p<0.01, *** p<0.001. β = adjusted regression coefficient; CI = confidence interval. Survey-adjusted subpopulation analysis.*

*†The "Other" category comprised approximately 19 officially recognized ethnic groups in Vietnam not represented by the 11 major ethnic groups.*

**Supplementary Table 2.** Factors associated with coexistence of stunting and wasting in children under six years of age (n = 7747)

|  | **Stunting and wasting** | | **Stunting, not wasting** | | **Wasting, not stuting** | |
| --- | --- | --- | --- | --- | --- | --- |
| **A. Household characteristics** |  |  |  |  |  |  |
| Household Economy |  |  |  |  |  |  |
| Poor | 1.52 | (0.91, 2.55) | 1.12 | (1.00, 1.26) | 0.96 | (0.66, 1.38) |
| Near-poor | 1.43 | (0.85, 2.42) | 1.15 | (0.97, 1.35) | 0.99 | (0.59, 1.68) |
| Better-off | 1 | (1.00, 1.00) | 1 | (1.00, 1.00) | 1 | (1.00, 1.00) |
| Households with ≥ 5 members | 1.13 | (0.85, 1.51) | 1.07 | (0.95, 1.22) | 0.98 | (0.81, 1.19) |
| Not using tap water | 1.36* | (1.03, 1.81) | 1.13 | (0.78, 1.63) | 1.15 | (0.82, 1.61) |
| Ownership of Toilet |  |  |  |  |  |  |
| No toilet | 1.06 | (0.64, 1.76) | 1.18* | (1.04, 1.34) | 1.06 | (0.82, 1.37) |
| Dry toilet (one or two tanks) | 1.58 | (0.80, 3.13) | 1.21** | (1.05, 1.40) | 0.82 | (0.53, 1.29) |
| Septic or semi-septic toilet | 1 | (1.00, 1.00) | 1 | (1.00, 1.00) | 1 | (1.00, 1.00) |
|  |  |  |  |  |  |  |
| **B. Maternal characteristics** |  |  |  |  |  |  |
| Mother age |  |  |  |  |  |  |
| 15-19 | 1.2 | (0.48, 3.00) | 0.83 | (0.67, 1.02) | 0.8 | (0.56, 1.14) |
| 20-24 | 1 | (1.00, 1.00) | 1 | (1.00, 1.00) | 1 | (1.00, 1.00) |
| 25-29 | 0.9 | (0.62, 1.31) | 0.9 | (0.80, 1.03) | 0.94 | (0.62, 1.42) |
| 30-34 | 1.11 | (0.66, 1.87) | 1.16* | (1.03, 1.31) | 1.02 | (0.74, 1.40) |
| 35-51 | 0.85 | (0.41, 1.77) | 0.96 | (0.80, 1.16) | 0.99 | (0.66, 1.49) |
| Mother ethnicities |  |  |  |  |  |  |
| Kinh | 1 | (1.00, 1.00) | 1 | (1.00, 1.00) | 1 | (1.00, 1.00) |
| Tay | 2.74 | (0.70, 10.69) | 1.14 | (0.84, 1.54) | 1.83 | (0.76, 4.41) |
| Nung | 2.07 | (0.42, 10.28) | 1.19 | (0.81, 1.76) | 1.12 | (0.34, 3.66) |
| Dao | 1.06 | (0.25, 4.52) | 1.52* | (1.08, 2.13) | 0.99 | (0.39, 2.52) |
| Hmong | 1.76 | (0.38, 8.23) | 2.74*** | (1.99, 3.78) | 0.74 | (0.28, 2.00) |
| Thai | 1.97 | (0.45, 8.62) | 1.27 | (0.79, 2.03) | 1.84 | (0.83, 4.07) |
| Ra Glai | 8.47** | (2.53, 28.36) | 2.08** | (1.30, 3.30) | 2.95* | (1.23, 7.06) |
| Mnong | 1.58 | (0.43, 5.80) | 1.03 | (0.81, 1.30) | 1.16 | (0.37, 3.69) |
| Co Tu | 1.21 | (0.28, 5.21) | 1.57 | (0.88, 2.80) | 0.78 | (0.31, 1.95) |
| Giay | 1.47 | (0.38, 5.72) | 1.37* | (1.00, 1.86) | 0.72 | (0.30, 1.72) |
| Khang | 1.55 | (0.30, 7.90) | 1.92** | (1.25, 2.94) | 1.61 | (0.20, 13.04) |
| Others† | 2.4 | (0.40, 14.52) | 1.53* | (1.07, 2.18) | 1.84 | (0.65, 5.18) |
| Mother education level |  |  |  |  |  |  |
| Primary level or below | 1.16 | (0.85, 1.58) | 1.26** | (1.06, 1.49) | 1.35* | (1.04, 1.74) |
| Junior high school level | 1 | (1.00, 1.00) | 1 | (1.00, 1.00) | 1 | (1.00, 1.00) |
| Highschool level | 1.5 | (0.79, 2.87) | 1.02 | (0.79, 1.31) | 1.48 | (0.93, 2.34) |
| Some college | 1.62 | (0.67, 3.88) | 0.81 | (0.61, 1.07) | 1.77 | (0.99, 3.16) |
| Occupation was a farmer | 1.63 | (0.85, 3.12) | 1.29** | (1.11, 1.49) | 1.26 | (0.82, 1.93) |
|  |  |  |  |  |  |  |
| **C. Child characteristics** |  |  |  |  |  |  |
| Antenatal checkup ≤3 times | 1.64 | (0.83, 3.21) | 1.29*** | (1.15, 1.43) | 1.22 | (0.84, 1.79) |
| Place of birth |  |  |  |  |  |  |
| Not in a health facility | 1.04 | (0.39, 2.78) | 1.12 | (0.92, 1.35) | 1.11 | (0.60, 2.07) |
| Commune health station | 0.95 | (0.65, 1.40) | 1.08 | (0.88, 1.32) | 1.13 | (0.67, 1.90) |
| Hospital | 1 | (1.00, 1.00) | 1 | (1.00, 1.00) | 1 | (1.00, 1.00) |
| Birthweight |  |  |  |  |  |  |
| Not recoreded | 1.07 | (0.47, 2.43) | 0.84 | (0.69, 1.04) | 0.66 | (0.40, 1.11) |
| < 2500 g | 2.58*** | (1.59, 4.21) | 1.52** | (1.13, 2.05) | 1.3 | (0.80, 2.10) |
| 2500–2999 g | 1 | (1.00, 1.00) | 1 | (1.00, 1.00) | 1 | (1.00, 1.00) |
| ≥ 3000 g | 0.33*** | (0.21, 0.51) | 0.58*** | (0.50, 0.67) | 0.61** | (0.46, 0.82) |
| The child is a boy | 1.64** | (1.16, 2.32) | 1.38*** | (1.26, 1.52) | 1.32 | (0.97, 1.80) |
| Child age |  |  |  |  |  |  |
| 0 | 1 | (1.00, 1.00) | 1 | (1.00, 1.00) | 1 | (1.00, 1.00) |
| 1 | 4.12*** | (2.67, 6.36) | 3.90*** | (2.87, 5.31) | 0.77 | (0.47, 1.26) |
| 2 | 2.55** | (1.46, 4.47) | 4.45*** | (3.04, 6.51) | 0.61*** | (0.47, 0.78) |
| 3 | 1.97 | (0.92, 4.20) | 3.43*** | (2.60, 4.52) | 0.45** | (0.26, 0.78) |
| 4 | 2.21* | (1.16, 4.22) | 2.91*** | (2.13, 3.95) | 0.33** | (0.18, 0.62) |
| 5 | 2.18* | (1.22, 3.88) | 2.36*** | (1.63, 3.42) | 0.74 | (0.47, 1.18) |

*Adjusted odds ratios (aORs) and 95% confidence intervals (95% CIs) were estimated using multinomial logistic regression, comparing each outcome category with children who were neither stunted nor wasted, and adjusting for the variables listed in the table. P‑values: *** < 0.001, ** < 0.01, * < 0.05.*

*†The "Other" category comprised approximately 19 officially recognized ethnic groups in Vietnam not represented by the 11 major ethnic groups.*
